# Expression pattern of immediate early genes in the cerebellum of D1R KO, D2R KO, and wild type mice under vestibular-controlled activity

**DOI:** 10.3389/fcell.2015.00038

**Published:** 2015-06-17

**Authors:** Toru Nakamura, Asako Sato, Takashi Kitsukawa, Toshikuni Sasaoka, Tetsuo Yamamori

**Affiliations:** ^1^Division of Brain Biology, National Institute for Basic BiologyOkazaki, Japan; ^2^Department of Basic Biology, Graduate University for Advanced Studies (SOKENDAI)Okazaki, Japan; ^3^KOKORO-Biology Group, Laboratories for Integrated Biology, Graduate School of Frontier Biosciences, Osaka UniversitySuita, Japan; ^4^Laboratory of Neurochemistry, National Institute for Basic BiologyOkazaki, Japan; ^5^Department of Laboratory Animal Science, Kitasato University School of MedicineSagamihara, Japan; ^6^Department of Comparative and Experimental Medicine, Brain Research Institute, Niigata UniversityNiigata, Japan; ^7^Laboratory of Molecular Analysis for Higher Brain Function, RIKEN Brain Science InstituteWako, Japan

**Keywords:** IEG, *c-fos*, *jun-B*, rota-rod, striatum

## Abstract

We previously reported the different motor abilities of D1R knockout (KO), D2R KO and wild-type (WT) mice. To understand the interaction between the cerebellum and the striatal direct and indirect pathways, we examined the expression patterns of immediate early genes (IEG) in the cerebellum of these three genotypes of mice. In the WT naive mice, there was little IEG expression. However, we observed a robust expression of *c-fos* mRNA in the vermis and hemisphere after running rota-rod tasks. In the vermis, *c-fos* was expressed throughout the lobules except lobule 7, and also in crus 1 of the ansiform lobule (Crus1), copula of the pyramis (Cop) and most significantly in the flocculus in the hemisphere. *jun-B* was much less expressed but more preferentially expressed in Purkinje cells. In addition, we observed significant levels of *c-fos* and *jun-B* expressions after handling mice, and after the stationary rota-rod task in naive mice. Surprisingly, we observed significant expression of *c-fos* and *jun-B* even 30 min after single weighing. Nonetheless, certain additional *c-fos* and *jun-B* expressions were observed in three genotypes of the mice that experienced several sessions of motor tasks 24 h after stationary rota-rod task and on days 1 and 5 after rota-rod tasks, but no significant differences in expressions after the running rota-rod tasks were observed among the three genotypes. In addition, there may be some differences 24 h after the stationary rota-rod task between the naive mice and the mice that experienced several sessions of motor tasks.

## Introduction

We have recently reported the distinct motor impairments of congenic dopamine D1 and D2 receptor KO mice in their performance of three different types of motor behavioral tasks, that is, spontaneous motor activity tasks in the home cage, rota-rod tasks, and our own invented Step-Wheel task (Kitsukawa et al., 2011). In the Step-Wheel task, mice run as they adjust their steps on foothold pegs to drink water (Nakamura et al., 2014). Durieux et al. (2012) showed that the ablation of D1R medium spiny neurons (MSNs) in the dorsolateral striatum (DLS) of mice corresponding to the primate putamen decreases the learning curve in rota-rod tasks. They also showed that the ablation of D2R MSN in the dorsomedial striatum (DMS) of mice corresponding to the primate caudate decreases the initial learning curve of the task. These results of local ablation are generally consistent with those of the entire striatal ablation of D1R and D2R neurons (Durieux et al., 2012) and the results of our behavioral experiments using general D1R and D2R KO mice, suggesting that DLS and DMS play major roles in rota-rod tasks. However, there are also differences in the results between local ablation and general D1R KO and D2R KO mice (Nakamura et al., 2014). The ablation of D1R MSNs in either DMS or the entire striatum reduced locomotion in the open field, which behavior is the reverse of general D1R KO mice. The ablation of D2R MSNs in either DMS or the entire striatum does not affect the late-phase performance (Durieux et al., 2012), whereas the general D2R KO mice showed poorer leaning than WT mice in the Step-Wheel tasks (Nakamura et al., 2014). These differences between local ablation of DRs and general KO suggest that the involvement and interaction between the striatum and other brain regions.

To explore this possibility further, in this study we employed the immediate early gene (IEG) mapping technique, with which the neuronal activity in the cerebellum is monitored on the basis of IEG expressions. Among eight IEGs we examined, *c-fos* and *jun-B* showed marked expression and specific induction, respectively, in the cerebellum immediately after the running rota-rod tasks. Interestingly, by examining *c-fos* and *jun-B* expressions in the vermis, Crus1, Cop and the flocculus in the cerebellar hemisphere, we found significant expressions of *c-fos* and *jun-B* 24 h after the stationary rota-rod tasks, during which time the mice did not perform any motor tasks but were allowed to move freely. We consider that this may be consistent with the idea that motor sequence learning and adaptation progress with time, through “consolidation” and “automatization” (Doyon et al., 2009).

We therefore examined in detail the expression patterns of *c-fos* and *jun-B* in the congenic D1R KO, D2R KO, and WT mice after the stationary and running rota-rod tasks in order to determine how cerebellar activities related to the D1R- and D2R-dependent pathways. Although we have not confirmed the alteration of *c-fos* expression patterns in the cerebellar flocculus on days 1 and 5 after the rotating rota-rod tasks among the three genotypes of mice, D1R KO, D2R KO, and WT mice, we found significant expressions of *c-fos* and *jun-B* not only immediately (30 min) after the mice ran on the rota-rod but also after handling them, and 30 min and unexpectedly even 24 h after the stationary rota-rod task. This observation led us to examine a series of examination for *c-fos* and *jun-B* expression patterns. We found that vestibular-controlled activities such as handling mice and weighing caused significant *c-fos* and *jun-B* expressions in cerebellar flocculus. However, there may be still some differences in expressions 24 h after the stationary rota-rod task between the naive and trained mice. Our results indicate that the analysis of *c-fos* and *jun-B* expression patterns is a useful tool for studying cerebellar activity, which may play a role complementary to imaging studies such as functional MRI, because of the fine resolution at the cellular level, in understanding the sequential events of motor learning in the cerebellum.

## Materials and methods

The expression of eight IEGs was first examined in the cerebellum of naive WT mice, that is, mice that did not experience any motor tasks but were allowed to move freely. The IEG expression patterns were examined in the mice that performed a rota-rod task (Experiment 1). Next, we compared *c-fos* and *jun-B* mRNA expression levels, in the cerebellar hemisphere, especially the flocculus among the three genotypes of mice: D1R KO, D2R KO, and WT mice (Experiment 2). Additionally, we examined the expressions patterns of *c-fos* and *jun-B* in the mice 30 min and 24 h after the stationary rota-rod tasks and 1-week handling.

For Experiment 1, adult male mice (8–9 week old, *n* = 5, C57BL/6J, Charles river laboratories Japan Inc.) were purchased. For Experiment 2, mice lacking either D1R (*n* = 7) or D2R (*n* = 9) were generated in accordance with the protocol previously published (Yamaguchi et al., 1996; Tran et al., 2008) and backcrossed for up to 10 generations with C57BL/6J (CLEA Japan Inc.) mice. Their genotypes were determined by PCR analysis of genomic DNA extracted from the tail of each mouse. As a control, C57BL/6J WT mice were purchased. All mice were housed individually in a plastic cage under 12 h light/dark cycle (9:00–21:00) and given food (Rodent Diet CA-1, CE-2, CLEA Japan Inc.) and water *ad libitum*. To encourage food intake to maintain their health (Drago et al., 1994; McNamara et al., 2003), D1R KO mice were additionally given palatable food (Rodent Diet B-F, CLEA Japan Inc.) on the floor of their cages. All the experiments were performed in accordance with the guidelines of the National Institutes of Health and the Ministry of Education, Culture, Sports, Science and Technology (MEXT) of Japan, and were approved by the Institutional Animal Care and Use Committees of the National Institutes of Natural Sciences, Kitasato University School of Medicine, and Frontier Biosciences of Osaka University. We made all efforts to minimize the number of animals used and the incidence or severity of distress experienced by the animals.

### Motor behavior tasks

In Experiment 1, WT naive mice were initially group housed for 1 week until they were segregated and individually housed during motor task training and testing as described in the Results. In Experiment 2, the same mice that experienced series of two types of motor tasks (rota-rod and Step-Wheel tasks) over a period of 4 months as described previously were used (Nakamura et al., 2014, detailed history is shown in **Figure 3** and Supplementary chart 1). In these series of experiments, the mice were divided into two groups for the rota-rod tasks: fast-slow (15–5 rpm) and slow-fast (5–15 rpm) groups (Nakamura et al., 2014). The mice were sacrificed at 10 months of age. We examined the expression patterns of *c-fos* and *jun-B* in the WT mice that did not previously experience any motor task at 30 min (*n* = 3) and 24 h (*n* = 3) after the stationary (0 rpm) rota-rod task. The rota-rod apparatus (MK-660A, Muromachi Kikai Co., Ltd.) was used. The rod diameter was 3 cm and the rotation speed was 10 rpm (Experiment 1) or 5 rpm (Experiment 2). In the running rota-rod tasks, a trial was considered when any one of the following three events occurred: The mouse (1) fell, (2) remained on the rod up to 60 s (Experiment 1) or 120 s (Experiment 2), or (3) clung to the rod for two complete turns, in which the mouse was considered to have fallen. The duration (retention times) that the mouse remained on the rod was recorded as the score. Five trials (Experiment 1) or three trials (Experiment 2) per day were performed and the interval between the trials was set at 30–60 s, during which time, the mice were placed in their home cages. Presessions consisted of trials for 1 day (Experiment 1) or 3 days (Experiment 2), during which the mice were placed on a stationary rod for habituation to the apparatus. Following the presessions, the running experiments were started. In Experiment 1, the mice were sacrificed 30 min after the last trial on day 1 (*n* = 2). In Experiment 2, the mice were divided into 3 groups: Pre (WT, *n* = 2; D1R KO, *n* = 2; D2R KO, *n* = 3), day 1 (WT, *n* = 3; D1R KO, *n* = 3; D2R KO, *n* = 3), and day 5 (WT, *n* = 3; D1R KO, *n* = 2; D2R KO, *n* = 3) groups. The mice of the Pre group were sacrificed on day 1, that is, the next day after the presessions (24 h after riding on the stationary rod). For the days 1 and 5 groups, the mice were sacrificed 30 min after the last trial. The rota-rod tasks were performed during the light phase (13:00-19:00). For this series of experiments, we weighed 30 min before the decapitation except for naive mice in which no weighing was done at least for a week before sacrifice.

### Handling

To investigate the effects of handling, WT mice (*n* = 2) that did not experience any motor tasks were handled for 1 week. The mice were placed on the palm of an experimenter for about 3 min per day. On the last day, the mice were decapitated 30 min after they were handled. On the other hand, the naive mice (*n* = 2) were fed *ad libitum*, and exposed to external factors as minimally as possible including experimenters and immediately decapitated.

### *In situ* hybridization (ISH)

*In situ* hybridization (ISH) was performed as described previously (Schaeren-Wiemers and Gerfin-Moser, 1993; Liang et al., 2000; Komatsu et al., 2005) with some modifications. The mice were quickly decapitated. The brain was removed, embedded in O.C.T. compound (Sakura) and quickly frozen in isopentane cooled with liquid nitrogen. Sections (10 μm thick) were cut on a cryostat (CM3050, Leica), thaw-mounted on slides (MAS coated glass slides, Matsunami), and air-dried. The sections were fixed in 4% paraformaldehyde (PFA) in 0.1 M phosphate buffer (PB, pH 7.3) for 15 min, washed three times in PBS, pH 7.4, acetylated for 10 min in 0.25% acetic anhydride in 0.1 M triethanolamine-HCl, pH 8.0, and washed three times with PBS. Prehybridization was performed using hybridization buffer [50% formamide, 5 × SSC (20 × SSC in 3 M NaCl, 0.3 M sodium citrate, pH 7.0), 5× Denhardt's, 250 μg/ml yeast tRNA, and 500 μg/ml salmon sperm DNA] for 30 min. DIG-labeled cRNA probes were denatured for 2 min at 82°C and chilled on ice. The sections were covered with hybridization buffer containing 1 μg/ml of the cRNA probes, added dropwise, and coverslipped. The information of each probes used in this study is shown in Supplementary Table 1. The slides were incubated overnight (14–18 h) at 72°C in a humidified chamber (50% formaldehyde, 5 × SSC), washed three times with 0.2 × SSC at 72°C, and then rinsed with TBS, pH 7.5 (100 mM Tris-HCl, pH 7.5, 150 mM NaCl). To detect the hybridized probes, the sections were blocked with 1× blocking solution (Roche) in TBS, pH 7.5, for 30 min, and then incubated in the blocking solution with an alkaline phosphatase (AP)-conjugated anti-DIG antibody (1:1000 dilution, Roche) overnight at 4°C. The sections were rinsed three times with TBS, pH 7.5, and equilibrated in TBS, pH 9.5 (100 mM Tris-HCl, pH 9.5, 100 mM NaCl, 50 mM MgCl_2_). Enzymatic activity was visualized by staining with 0.2 mM 5-bromo-4-chloro-3-indolyl-phosphate, 0.2 mM nitroblue tetrazolium (NBT/BCIP) in TBS, pH 9.5, in the dark, until the signal reached a satisfactory intensity. After a brief wash in PBS and distilled water, the sections were dehydrated in a series of gradually increasing concentrations of ethanol solutions. After immersion in xylene, the sections were mounted in Entellan, a new rapid mounting medium (Merck).

## Results

### IEG expression patterns in the cerebellum

**Figure 1:**
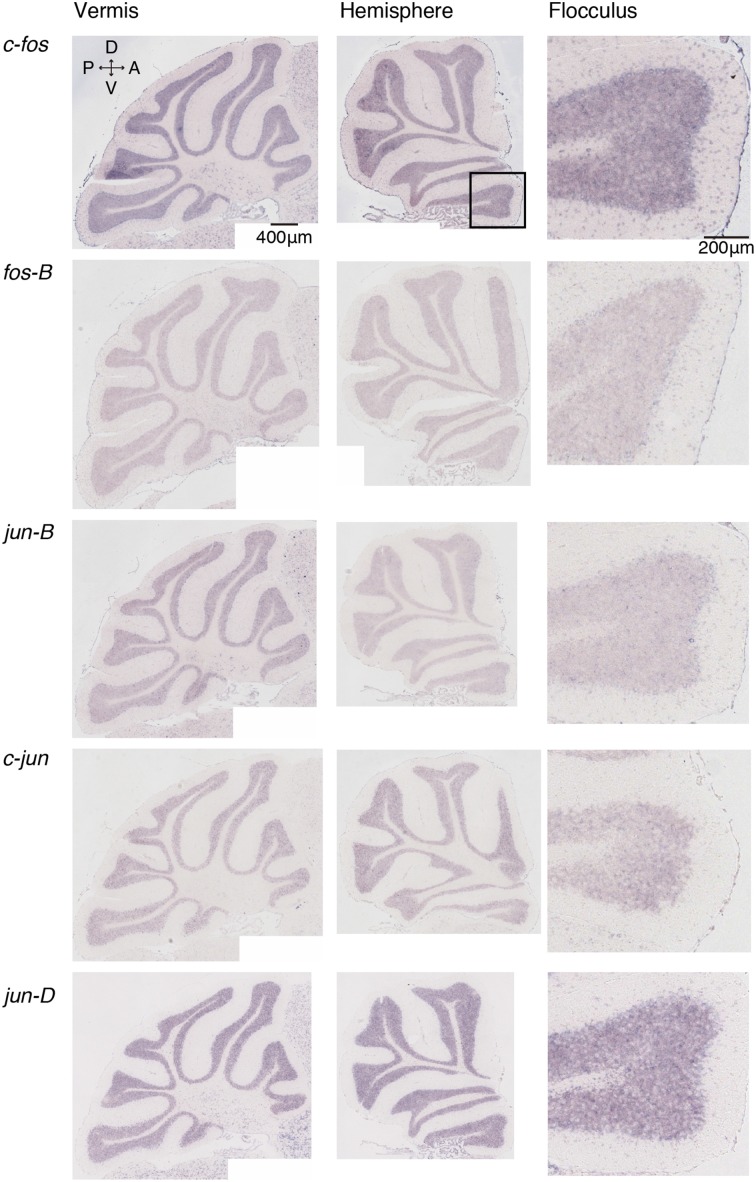
**Expression patterns of eight IEGs in the cerebellum of naive mice**. Vermis (**left panel**), hemisphere (**middle panel**), and the magnified image within the box in the middle panel showing flocculus (**right panel**). Each row shows expression patterns of eight IEGs (from top to bottom: *c-fos, fos-B, jun-B, c-jun, jun-D*).

**Figure 2:**
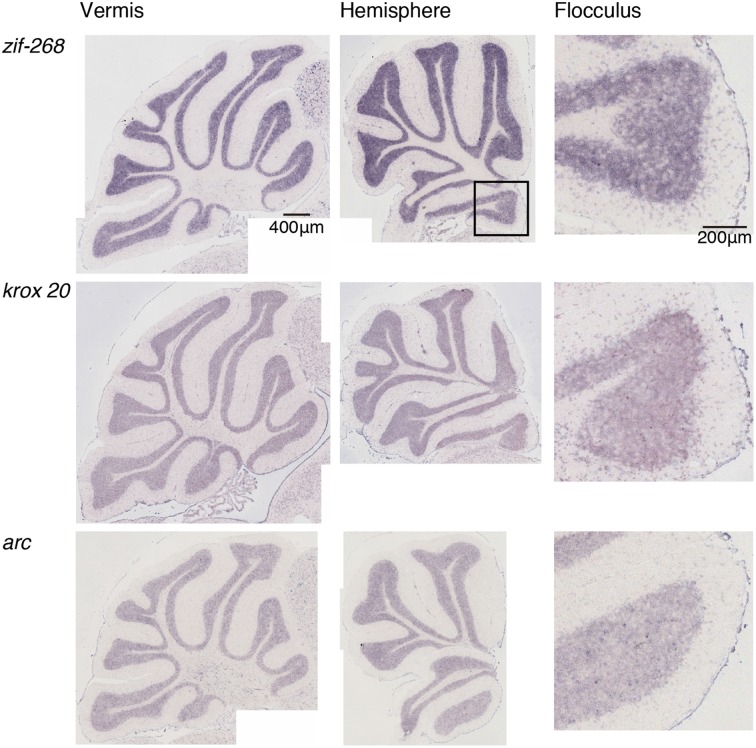
**Expression patterns of eight IEGs in the cerebellum of naive mice**. Vermis (**left panel**), hemisphere (**middle panel**), and the magnified image within the box in the middle panel showing flocculus (**right panel**). Each row shows expression patterns of eight IEGs (from top to bottom: *zif-268, krox 20, arc*).

**Figure 3:**
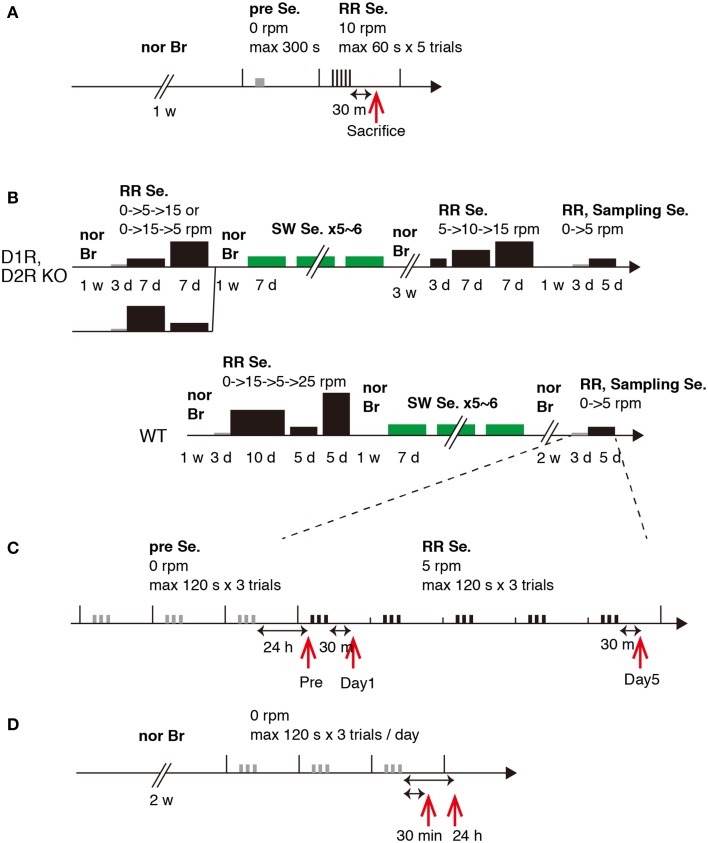
**Experimental schedules**. Schematic experimental procedures for Experiments 1 and 2. **(A)** Experiment 1. **(B)** Experiment 2, WT, D1R KO, and D2R KO mice, training schedule. **(C)** Experiment 2, sampling schedule. **(D)** Experiment 2, 30 min and 24 h after stationary rota-rod task. Abbreviations: nor Br, normal breeding; Se., session group; RR, rota-rod; SW, Step-Wheel; w, week; d, day; h, hour; m, minute; s, second. The width and height of the black bar represent task time and speed of rota-rod, respectively.

**Figure 4:**
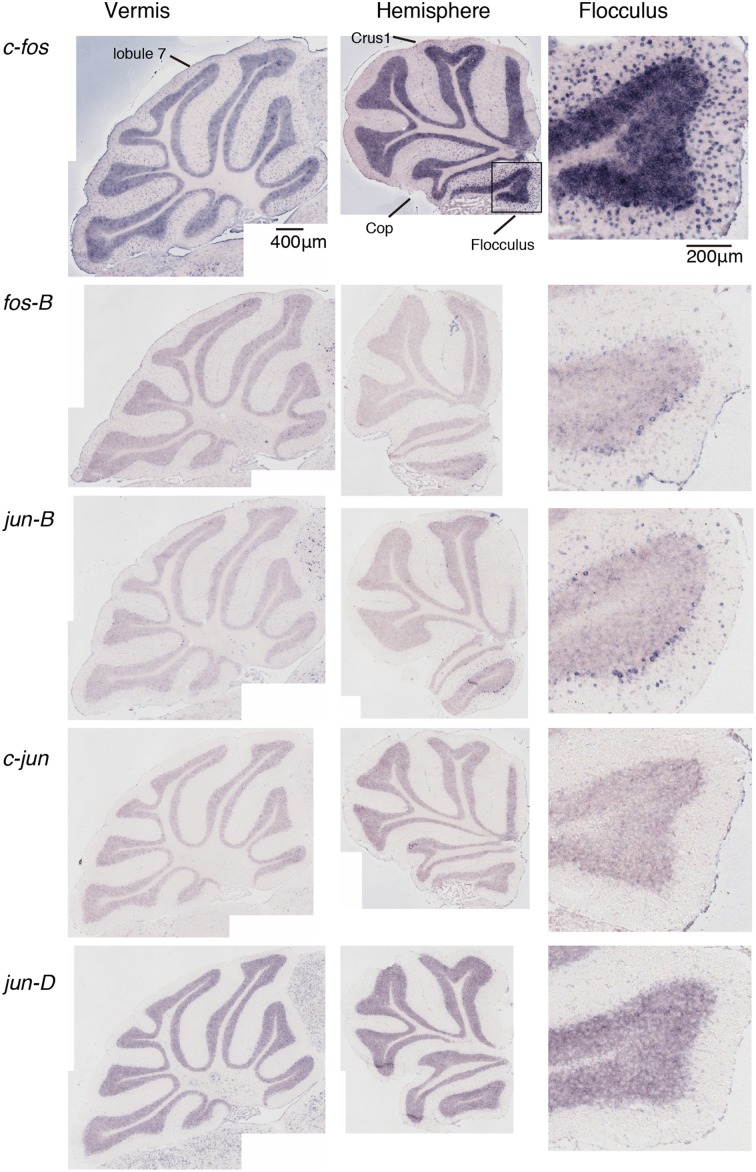
**Expression patterns of eight IEGs in the cerebellum of mice that performed rota-rod tasks**. IEG expression patterns in the mice that performed three trials of the rota-rod tasks (see Materials and Methods). The figures are lined up in the same order as in Figure 1.

**Figure 5:**
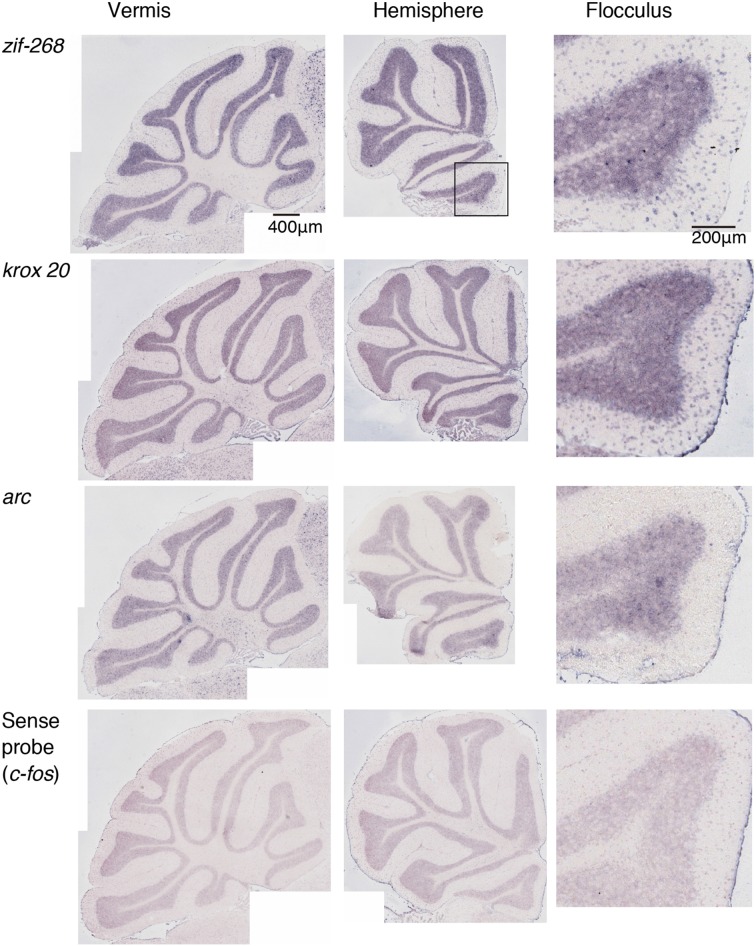
**Expression patterns of eight IEGs in the cerebellum of mice that performed rota-rod tasks**. IEG expression patterns in the mice that performed three trials of the rota-rod tasks (see Materials and Methods). The figures are lined up in the same order as in Figure 2. The last (4th) row shows the signals hybridized with a sense probe of *c-fos*, indicating the background level.

We examined the IEG expression patterns in the cerebellum. In our previous study, we examined the expression patterns of IEGs including the *fos* and *jun* families in cerebellar slices without and with treatment with α-amino-3-hydroxy-5-methyl-4-isoxazole-propionate (AMPA) and/or 8-bromo-cGMP (8-Br-cGMP) (Nakazawa et al., 1993). That study showed that untreated slices express low levels of IEGs but slices treated with AMPA and 8-Br-cGMP express high levels of *c-fos*, *jun-B* and *zif-268* (*NGFI-A)*. Following that study, we first examined IEG expression patterns in the cerebellum. Naive mice that were moving freely but doing no specific tasks were sacrificed, and the IEG expression patterns of the *fos* family (*c-fos*, *fos-B*), *jun* family (*jun-B*, *c*-*jun*, and *jun-D*), and other IEGs of *zif-268* (*egr 1*, *NGFI-A*), *krox 20* (*egr 2*, *NGFI-B*), and *arc* were examined immediately after the sacrifice (see Materials and Methods). Consistent with the previous results, we observed little expression of these eight IEGs in the cerebellum of naive mice (Figures 1, 2). We next examined the expression patterns of these IEGs immediately after the mice performed five trials of running rota-rod tasks (Figure 3A). The mice were sacrificed 30 min after the last trial and the IEG expression patterns in the cerebellum were examined (Figures 4, 5). Among the members of the *fos* and *jun* families, *c-fos* was expressed in almost all the lobules of the vermis (except lobule 7), and Crus1, Cop and most significantly in the flocculus in the hemisphere. It is strongly expressed in the granular and Purkinje layers, and some intense signals were observed in the molecular layer. *jun-B* was also expressed in the same regions as *c-fos* was expressed but more preferentially in Purkinje cells and some sparsely scattered intense signals of *jun-B* were observed in the molecular layer. We observed some weak expression of *fos-B* in the flocculus as well. In the cerebellar slices, although we previously observed significant *zif-268* expression (Nakazawa et al., 1993), here we observed only a generally moderate expression of *zif-268* under the present condition. *krox 20* was also expressed to some extent in the flocculus. Note that the adjacent external layer of the inferior colliculus expressed *arc* and *zif-268* at certain levels, which may be used as internal controls for evaluating the IEG expression levels in the cerebellum. Overall, *c-fos* and *jun-B* among the IEGs examined in the flocculus showed most prominent expressions immediately (30 min) after the rota-rod running tasks. Therefore, hereinafter, we focused on the expression patterns of *c-fos* and *jun-B* in the flocculus.

### *c-fos* and *jun-B* expression patterns in stationary rota-rod task in naive mice

As we mentioned in Introduction, we were interested in the functional relationship between the cerebellum and the striatum and explored a model experimental system. Dopamine receptors are distributed in certain brain regions (Gingrich and Caron, 1993). However, our previous studies demonstrated that D1R KO and D2R KO mice showed distinguished motor impairments (Nakamura et al., 2014). In the running rota-rod tasks, the impairments were largely similar to those resulting from local ablation of D1R neurons and D2R neurons in DLS and DMS (Durieux et al., 2012), respectively. These results suggest that when using D1R KO and D2R KO mice in the rota-rod tasks, the main target is the striatum. We therefore used D1R KO and D2R KO mice to examine the cerebellar flocculus IEG (*c-fos* and *jun-B*) expression patterns and the relationship between the striatum and the cerebellum in relation to motor learning.

**Figure 6:**
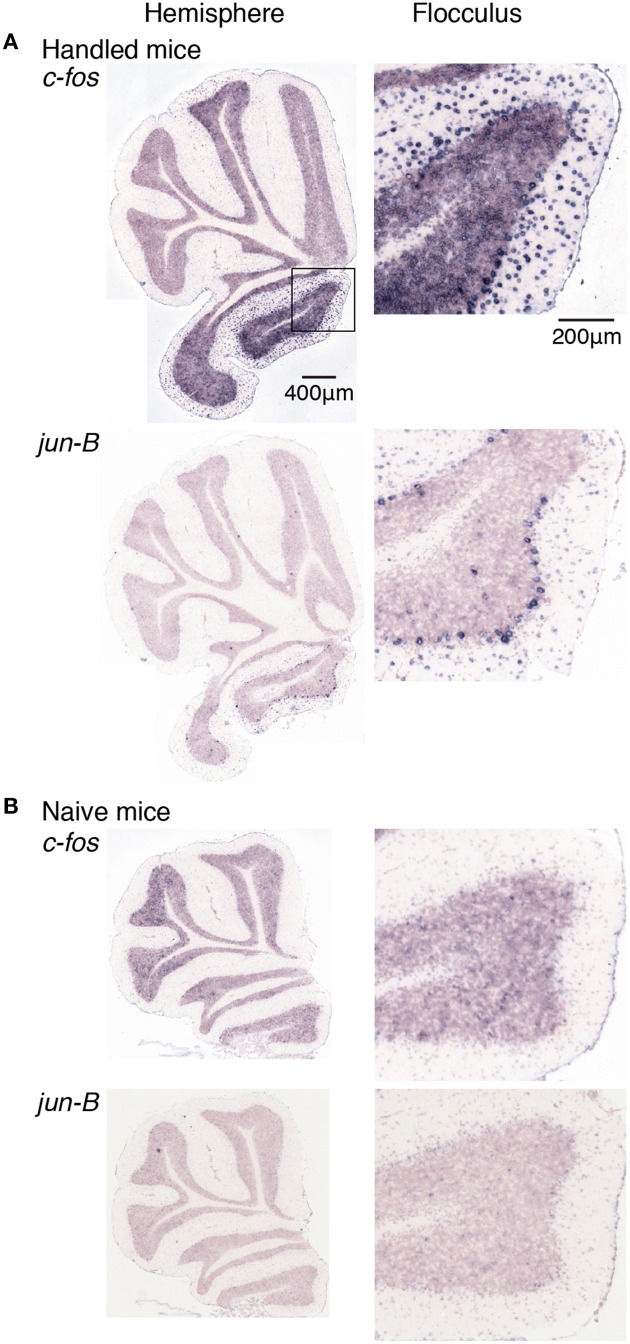
**Expression levels of *c-fos* and *jun-B* mRNAs in the cerebellum of mice handled for a week**. **(A)** Mice handled for a week (see Materials and Methods). **(B)** Naive mice that were allowed to move freely but were not handled by humans. Sections of the hemisphere that showed the highest signal intensity for each gene are shown (left panel), Right panels show magnified images of floccular signals enclosed in a box in the top image of the left panel.

**Figure 7:**
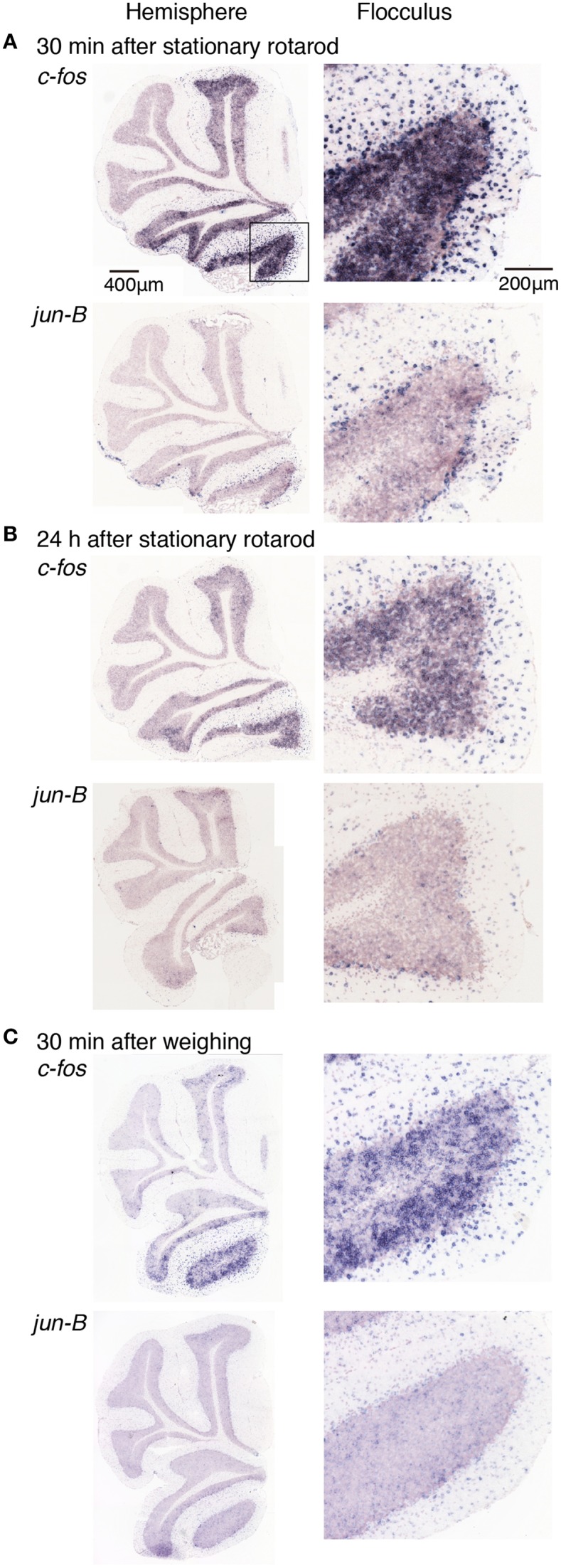
**Expression levels of *c-fos* and *jun-B* mRNAs in the cerebellum of mice after stationary rota-rod task or single weighing**. Mice that did not experience any motor tasks (untrained) were sacrificed **(A)** 30 min, **(B)** 24 h after the stationary rod task (see Materials and Methods), or **(C)** 30 min after single weighing. Right panels show magnified images of floccular signals.

**Figure 8:**
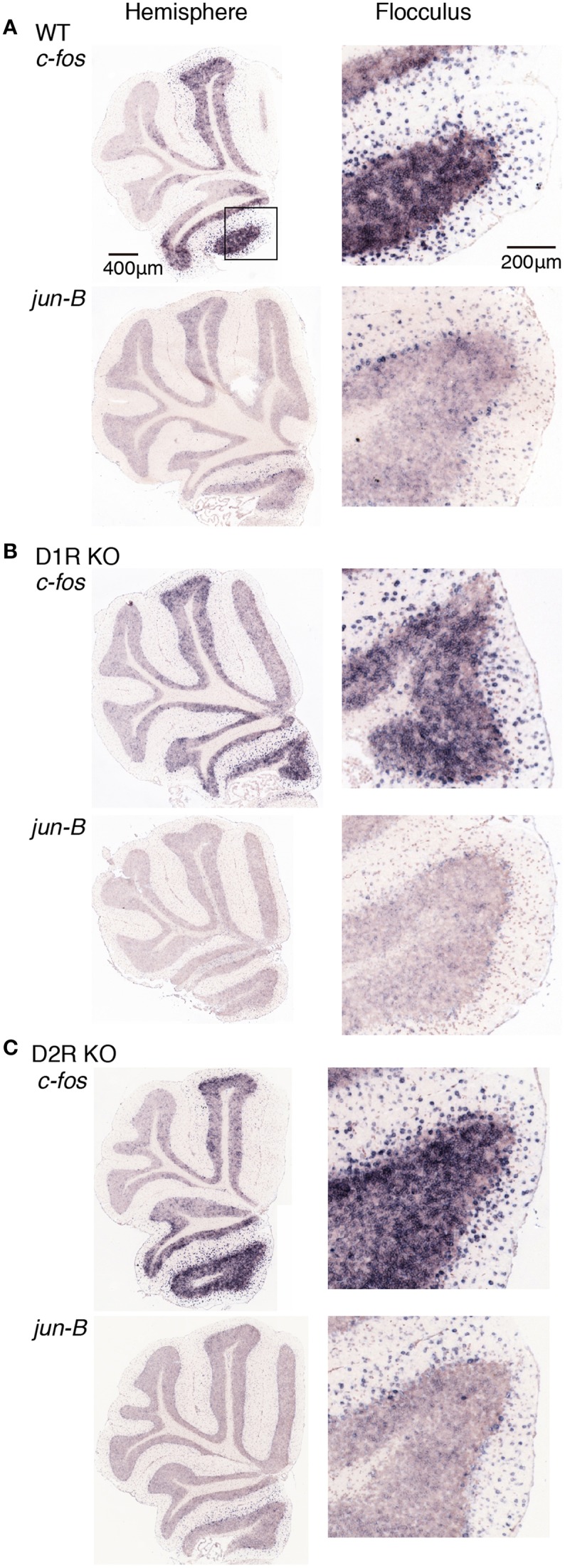
**Expression levels of *c-fos* and *jun-B* mRNAs in the cerebellum of trained mice after riding on stationary rota-rod**. **(A)** WT, **(B)** D1R KO, and **(C)** D2R KO mice were trained to perform a series of rota-rod and Step-Wheel tasks (see Materials and Methods). These mice were sacrificed 24 h after the stationary rota-rod task and designated as the Pre group for the running rota-rod tasks (see Materials and Methods). The left panels show the sections from the hemisphere that showed the highest signal intensity among the sections examined in each row. The right panels show the magnified images of the flocculus enclosed in a box in the first row of the left panel.

**Figure 9:**
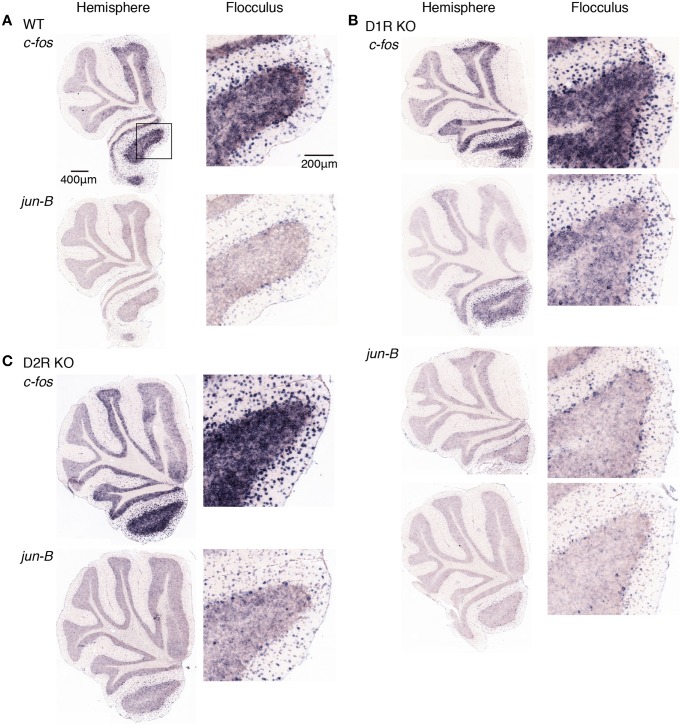
**Expression patterns of *c-fos* and *jun-B* mRNAs in the cerebellum of trained mice after rota-rod task on day 1**. **(A)** WT, **(B)** D1R KO, and **(C)** D2R KO mice were trained to perform a series of rota-rod and Step-Wheel tasks. After the presessions (0 rpm, 3 days), the mice performed rota-rod tasks (5 rpm) and were sacrificed 30 min after the session on the day 1. The left panels show the sections from the hemisphere, the right panels show magnified images of the part enclosed in a box in the first row of the left pane that shows the flocculus. **(B)** D1R KO mice, the top row shows *c-fos* expression of the mice under the fast-slow (15–5 rpm) condition at the training sessions, and the second row shows that under the slow-fast (5–15 rpm) conditions (see Materials and Methods). Bottom two rows show *jun-B* expressions in this order. **(C)** D2R KO mice, on the day 1 after a rotating rota-rod task (5–15 rpm). The 15-5 rpm task was not done for this group of mice (D2R KO, day 1).

**Figure 10:**
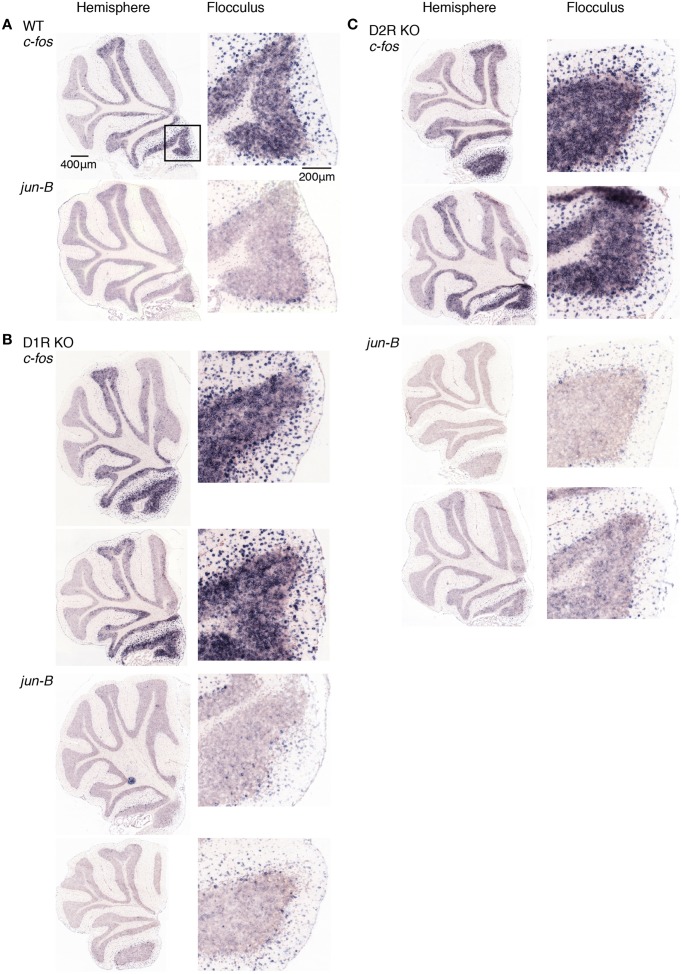
**Expression patterns of *c-fos* and *jun-B* mRNAs in the cerebellum of trained mice after rota-rod task on day 5**. **(A)** WT, **(B)** D1R KO, and **(C)** D2R KO mice were trained to perform a series of rota-rod and Step-Wheel tasks. After Pre sessions (0 rpm, 3 days), mice performed rota-rod tasks (5 rpm) and were sacrificed 30 min after the session on day 5. The left panels show the sections from the hemisphere, the right panels show magnified images of the flocculus enclosed in a box in the top image of the left panel, which shows the flocculus. **(B)** D1R KO and **(C)** D2R KO mice. Top row shows the sections from mice under fast-slow (15–5 rpm) conditions during the training sessions, and the second row shows the sections from mice under slow-fast (5–15 rpm) conditions (see Materials and Methods for details).

We first examined the expression patterns of *c-fos* and *jun-B* in the stationary rota-rod task. In this task, a mouse was placed on a non-rotating bar with a thickness about half the length of the mouse's body. This task was not too difficult for mice to perform, and not only WT mice but also D1R KO and D2R KO mice were able to stay for the time examined (Supplementary Figure 2). Nonetheless, this task requires mice to balance on the bar and therefore activates related brain regions. In parallel, we first examined the *c-fos* and *jun-B* expression patterns in the cerebellum of WT mice after they were handled for 1 week (Figure 6). Herein, from Figures 6–10 for all the ISH data, we examined 6–13 sections of the cerebellums of individual mice under each motor condition as described for each figure. We examined two to five sections of the vermis and four to nine sections of the hemisphere in each mouse, and here used the sections that showed the highest signal intensity of the IEG expression in the flocculus among the sections of the cerebellum under each condition. We were surprised to find high expression levels of *c-fos* and *jun-B* in the cerebellum immediately (30 min) after handling (Figure 6). In the hemisphere, we found moderately high expression levels in Crus1, and Cop and high expression levels in the flocculus. The expressions of *c-fos* and *jun-B* were probably induced by the attempts of mice to maintain their balance on the palm of the hand during the handling. The expression patterns of *c-fos* and *jun-B* appeared to be overall similar between the conditions (handling, stationary and running rota-rod), although there may have been some differences. We also found the expression of these genes not only immediately (30 min) after but also 24 h after the stationary rota-rod task for WT naive mice (Figures 3D, 7A,B) and for the same duration in the D1R KO, D2R KO, WT trained mice (Figures 3B,C, 8, three sessions as the trained mice). The expressions of *c-fos* and *jun-B* in the flocculus even 24 h after the stationary rota-rod task was unexpected because IEG expressions would not last for such a long time. We weighed the mice 30 min prior to increase their sacrifice 24 h after the stationary rota-rod task. The weighing involved placing the mice on the scales of a balance, which may have activated the vestibular systems. It was indeed the case because we observed significant expression of *c-fos* and *jun-B* 30 min after only single weighing (Supplementary Figure 3A). We also examined the expression patterns of these two IEGs 24 h after the stationary rota-rod task in the mice that experienced several sessions of the wheel running and rota-rod tasks (rotating rota-rod and Step-Wheel tasks, see Materials and Methods for details, Figure 8). In the naive mice, which only experienced the stationary rota-rod task, the expression levels of *c-fos* and *jun-B* were reduced to some extent but were still significant. In the mice that experienced several sessions of motor tasks, the expressions, in particular that of *jun-B*, appeared to be more sustained than those in the naive mice (Figures 7, 8). We also noticed that the expression pattern of *c-fos* and *jun-B* may be different between those of 30 min after only weighing in naive mice and 24 h after stationary rota-rod in trained mice (Supplementary Figures 4, 5).

### *c-fos* and *jun-B* expression patterns in stationary and rotating rota-rod-tasks in trained mice

Doyon et al. (2009) proposed that the basal ganglia are functionally related to motor learning. They propose that motor sequence learning and adaptation progress with time through the “consolidation” and “automatization” processes. During the course of these processes, the basal ganglia, cerebellum, hippocampus and cerebral cortex interact with each other, and, with time, the sites of plasticity are shifted to the cerebral cortex and striatum for motor sequence learning and the cerebral cortex and cerebellum for motor adaptation. We thus examined the cerebellar IEG expression patterns 24 h after the stationary rota-rod task in the mice that experienced several motor tasks (rotating rota-rod and Step-Wheel tasks, see Materials and Methods for details) in order to determine whether there are any differences in cerebellar activity among the three different genotypes of mice immediately (30 min) after they performed running rota-rod tasks on days 1 and 5 (Figures 9, 10).

Figures 9, 10 show the expression patterns of *c-fos* 24 h after the stationary rota-rod task in the three genotypes of mice after the rota-rod tasks on days 1 and 5, respectively. We observed significant expressions of *c-fos* in the hemisphere even 24 h after the stationary rota-rod task. Among the regions in the hemisphere, the expression in the flocculus was most significant. The *c-fos* expression is generally similar between days of 1 and 5. Figures 9, 10 show cerebellar *jun-B* expression patterns in WT mice. *jun-B* was expressed in the cerebellar vermis, and Crus1 and Cop of cerebellar hemisphere. Although *jun-B* was expressed to some extent in the molecular layer and at a lower level in the granular layers, the expression was most prominent in Purkinje cells. *jun-B* expression in the Purkinje cells in the flocculus seemed to be similarly enhanced in the trained mice 24 h after the stationary rota-rod task in contrast to the naive mice, and immediately after the running rota-rod tasks on days 1 and 5.

D1R KO, D2R KO, and WT mice expressed *c-fos* in the cerebellar hemisphere, particularly in flocculus 24 h after the stationary rota-rod tasks and on days of 1 and 5 after the rotating rota-rod tasks. The expression levels of *c-fos* and *jun-B* 24 h after the stationary rota-rod task in D1R KO mice appeared lower than those in D2R KO and WT mice, but since we failed in the staining of ISH in one of D1R KO mice and we were unable to make a conclusion on the significance of this finding. The expression of *c-fos* in the granular layer of the flocculus of D2R KO mice might be more enhanced on day 1 after the running rota-rod tasks than under the other conditions (Figures 8–10). *jun-B* expression in D2R KO mice was also enhanced 24 h after stationary rota-rod and on days 1 and 5, and may be more enhanced on day 1. These observation, however, were not statistically confirmed any further owing to the lack of a sufficient number of mice of these three genotypes available for series of motor tasks.

## Discussion

We examined cerebellar expression patterns of eight different IEGs in D1R KO, D2R KO, and WT mice following stationary and rotating rota-rod tasks. *c-fos* expression was enhanced in all the lobules of the vermis except lobule 7 and in Crus1, Cop and the flocculus in the hemisphere, whereas *jun-B* expression was only enhanced in some of the Purkinje cells in these regions. Furthermore, in this study we surprisingly found that *c-fos* and *jun-B* in the cerebellar vermis and flocculus were significantly expressed in mice after handling and 24 h after the stationary rota-rod task. We also found robust expressions of *c-fos* and *jun-B* after the running rota-rod tasks on days 1 and 5 in D1R KO, D2R KO, and WT mice.

### IEG expression patterns in the cerebellum

We examined the IEG expression patterns in the cerebellum. Among the eight IEGs we examined, *c-fos* was significantly expressed in all lobules of the cerebellar vermis except lobule 7 and in Crus1, Cop and the flocculus of the cerebellar hemisphere. *jun-B* was preferentially expressed in the Purkinje cells in these regions. *arc* is known to be induced by various types of neural activities including visual stimulation (e.g., Nakagami et al., 2013); there is virtually no induction in the cerebellum (Figure 5). *zif-268* is also known to be induced upon various neural activation, but was little induced in the cerebellum (Figure 5). One plausible reason why *arc* and *zif-268* are not induced in the cerebellum is that cerebellar Purkinje cells do not express functional NMDA receptors (Perkel et al., 1990; Llano et al., 1991). For example, *arc* transcription is regulated by NMDA- or voltage-gated calcium channel (VGCC)- mediated membrane depolarization, cAMP-dependent pathways and brain-derived neurotrophic factor (BDNF)-mediated pathways (Zheng et al., 2009). However, the exact mechanisms underlying non-expression of arc in the cerebellum need to be elucidated in future studies.

*c-fos* mapping showed the regions activated immediately after the running rota-rod tasks. In the vermis, *c-fos* was moderately induced in all the lobules except lobule 7 throughout the cerebellar vermis. In the hemisphere, significant expressions of *c-fos* were observed in Crus1, Cop, and flocculus. This observation indicates that the cerebellar vermis plays a central role in visually guided movements (Edge et al., 2003) and receives limb and visual inputs (see Edge et al., 2003 for reference). Crus1 also receives visuomotor inputs (Edge et al., 2003; Cerminara et al., 2005). Hindlimb stimulation primary activates the medial aspect of Cop (Santori et al., 1986). Our *c-fos* mapping results in this report well match those of the previous studies that examined the cerebellar areas activated by visuomotor inputs. One new observation in *c-fos* mapping may be the neuronal activation in the flocculus in the cerebellar hemisphere after the stationary and running rota-rod tasks. This is probably because the rota-rod tasks required mice to maintain their balance, which likely activated eye movements and the vestibular systems. The vestibular-ocular reflex presumably activates the flocculus (Ito, 1982).

### *c-fos* and *jun-B* expression patterns after stationary rota-rod task

We first examined the *c-fos* and *jun-B* expression patterns in the cerebellum immediately and 24 h after the mice performed the stationary rota-rod task in which mice were required to stay on the non-rotating (stationary) rota-rod. This is not a very difficult task for mice to perform, and both D1R KO and D2R KO mice were able to stay as long as WT mice did (Supplementary Figure 2). However, to our surprise, even 24 h after performing the stationary rota-rod task, we observed significant *c-fos* and *jun-B* expressions in the cerebellum. It was puzzling why the cerebellum was activated 24 h after the task, during which time the mice did not perform any specific tasks. One possible explanation for this would be as follows: there were other brain regions that were activated, which in turn activated the cerebellum. Interaction among the cerebellum, basal ganglia and cerebral cortex has been proposed (Doya, 2000; Hikosaka et al., 2002; Doyon et al., 2009; Bostan et al., 2013). It is therefore possible that the cerebellum was activated by other brain areas such as the basal ganglia. We therefore examined the *c-fos* and *jun-B* expression patterns in D1R KO and D2R KO mice after the running rota-rod tasks. We weighed the mice 30 min before sacrifice after 24 h after stationary rota-rod tasks. The weighing involved placing the mice on the scales of a balance, which may have activated the vestibular systems. It was indeed the case as shown in Supplementary Figure 3. However, the enhancement of *c-fos* and *jun-B* 24 h after the stationary rota-rod task in the mice that experienced several sessions of the motor tasks seems to be higher than that of 24 h after the stationary rota-rod for naive mice (Figures 7, 8). We also noticed the different IEG expression pattern between after only single weighing in naive mice and 24 h after stationary rota-rod in trained mice although both groups of mice were sacrificed 30 min after the last weighing (Supplementary Figures 4, 5). This difference may reflect the shift of cerebellar activation by forming internal model within the cerebellum (Bursztyn et al., 2006).

The expression of *c-fos* did not appear to be altered between days 1 and 5 after the running rota-rod tasks in D1R KO, D2R KO, and WT mice. However, there may be some difference in *jun-B* expression under our conditions. Durieux et al. (2012) and Nakamura et al. (2014) demonstrated that D1R KO mice and, mice with complete ablation of D1R neurons in the striatum showed decreased learning curve and performance after learning of the rota-rod tasks, whereas mice with ablated D2R neurons in the DMS and the entire striatum showed decreased initial performance of the rota-rod tasks. It has been reported that cerebellar activity reflects an acquired internal model (Imamizu et al., 2000). At the time when the impairment of striatal functions is severe, the cerebellar region works to compensate for the impairment and will presumably be most activated.

The expression of IEG and the downstream regulated genes in the striatum of D1R KO, D2R KO mice has been reported upon pharmacological stimulation (Drago et al., 1996; Aoyama et al., 2000; Welter et al., 2007). In D1R KO mice cocaine did not induce *c-fos* and *zif-268* expressions in the striatum (Drago et al., 1996). In D2R KO mice cocaine did not induce *c-fos* but induce *zif-268* in the striatum (Welter et al., 2007). In the striatum D1R is coexpressed with substance P (SP) and D2R is coexpressed with encephalin (ENK). In D1R KO mice SP expression was reduced and ENK expression was unaltered. Cocaine induced SP but not ENK in the striatum of D1R KO mice. In D2 KO mice ENK expression was increased and SP expression was decreased (Aoyama et al., 2000). In D1R KO mice cocaine induced SP and but did not affect ENK expression (Drago et al., 1996). In D2 KO mice cocaine induced SP expression. Therefore, These previous studies indicated that by in large the direct (D1R) and indirect pathways (D2R) are affected as expected as we write in the Introduction in D1R KO and D2R KO mice in terms of the IEG expression. Compared to these previously reported results in the striatum where the D1R and D2R play direct roles in the regulation of IEGs, in the cerebellum there is little or only indirect effect in the IEG expression, if any, in D1R and D2R KO mice as we showed in this paper.

We have been using chemical methods for detecting ISH because of their high spatial resolution. However, for quantitative evaluation, chemical detection methods are not as quantitative as radioisotope detection methods, in which the amount of radiation is strictly proportional to that of radioactive decay. Various conditions could affect chemical reactions; therefore, exact quantitation is difficult without an internal control. In this regard, our visual evaluation of *c-fos* expression in the flocculus on days 1 and 5 after the running rota-rod tasks in D1R KO and D2R KO mice should be more reliable because it enabled us to compare the expression with that in other lobules of the same section of the cerebellar hemisphere as an internal control.

It should be noted that *jun-B* was preferentially expressed in Purkinje cells after the rota-rod tasks. This expression pattern is quite in contrast to that of *c-fos*, which was particularly highly expressed in the granular layer and other layers as well. We previously reported that *jun-B* is selectively induced by conjunctive stimulation of climbing fibers and AMPA in the cerebellum, which mimics cerebellar LTD (Yamamori et al., 1995). *jun-B* expression level in the mice 24 h after the stationary rota-rod task may be higher in the mice that experienced several motor tasks than in the naive mice. However, these apparent differences should be examined by statistical analysis with a larger number of mice in future studies.

### Implications of studies of *c-fos* and *jun-B* expression patterns in the cerebellum in D1R KO and D2R KO mice

In this study, we applied techniques of IEG mapping in the cerebellum to detect the altered cerebellar neuronal activity after the stationary and running rota-rod tasks. We summarized the expression patterns of *c-fos* and *jun-B* in the flocculus in the cerebellum in Table 1. We consider that there are three main points in our studies reported in this paper. First, we found several different levels of expressions of *c-fos* and *jun-B* in the cerebellum in various conditions of weighing and handling, 30 min and 24 h after the stationary rota-rod task, and 30 min after running rota-rod on days 1 and 5. There may be some enhancement of *c-fos* and *jun-B* 24 h after the stationary rota-rod in the trained mice even considering the weighing effect for the expressions of *c-fos* and *jun-B*. Second, to further examine the interaction between the cerebellum and the striatum, we examined the *c-fos* and *jun-B* expressions in the cerebellum in D1R KO, D2R KO, and WT mice after the stationary and running rota-rod tasks. We did not observe significant differences among the three genotypes although this does not still exclude the possibility that there are some differences among them. To clarify whether there are indeed differences among the three genotypes, a larger of number of mice need to be examined and statistical analysis should be conducted. Third, our results demonstrated the particular usefulness of *c-fos* and *jun-B* for examining expression patterns in the cerebellum, because other IEGs are little expressed in the cerebellum or not selectively expressed in Purkinje cells.

**Table 1:** ***c-fos* and *jun-B* expressions in cerebellar flocculus**.

	**WT**		**D1R KO**		**D2R KO**	
	***c-fos***	***jun-B***	***c-fos***	***jun-B***	***c-fos***	***jun-B***
Naïve	−	−	NT	NT	NT	NT
Handling (for 1 week)	++	++	NT	NT	NT	NT
Stationary RR 30 min	+++	++	NT	NT	NT	NT
Stationary RR 24 h after (for naive mice)	++	+	NT	NT	NT	NT
Stationary RR 24 h (for trained mice)	+++	++	++^*^	+^*^	+++	++
Running RR Day 1	+++	++	+++	++~+++^**^	+++	+++
Running RR Day 5	+++	++	+++	++	+++	++
Weighing	+~++^***^	+^***^	NT	NT	NT	NT

*Summary of the expression levels of c-fos and jun-B in the cerebellar flocculus are represented; −, background level; +, weak positive; ++, positive; +++, highly positive signal*.

* *Only one mouse and somewhat weaker staining than other samples*.

** *Some variations between two mice*.

*** *Uneven expression within floccullus. NT: not tested. Note that all animals were sacrificed 30 min after final task except for the naive mice that were sacrificed without weighing. The evaluation was done subjectively with agreement of by two of authors (Toru Nakamura and Tetsuo Yamamori)*.

### Conflict of interest statement

The authors declare that the research was conducted in the absence of any commercial or financial relationships that could be construed as a potential conflict of interest.
