# Supplementary material for: Expression pattern of immediate early genes in the cerebellum of D1R KO, D2R KO, and wild type mice under vestibular-controlled activity
Source: Front Cell Dev Biol. 2015 Jun 17;3:38. doi: 10.3389/fcell.2015.00038 (PMC4469897; doi:10.3389/fcell.2015.00038)
Supplement: Supplementary file 2 [file DataSheet3.DOCX]

***Supplementary Material***

**Expression pattern of immediate early genes in the cerebellum of D1R KO, D2R KO and wild type mice under vestibular-controlled activity**

**Toru Nakamura^1,2,3^, Asako Sato^4,5^, Takashi Kitsukawa^1,3^, Toshikuni Sasaoka^4,5,6^ and Tetsuo Yamamori^1,2,7*^**

^1^Division of Brain Biology, National Institute for Basic Biology, Okazaki, Japan

^2^Department of Basic Biology, Graduate University for Advanced Studies (SOKENDAI), Okazaki, Japan

^3^KOKORO-Biology Group, Laboratories for Integrated Biology, Graduate School of Frontier Biosciences, Osaka University, Suita, Japan

^4^Laboratory of Neurochemistry, National Institute for Basic Biology, Okazaki, Japan

^5^Department of Laboratory Animal Science, Kitasato University School of Medicine, Sagamihara, Japan

^6^Department of Comparative and Experimental Medicine, Brain Research Institute, Niigata University, Niigata, Japan

^7^Laboratory of Molecular Analysis for Higher Brain Function, RIKEN Brain Science Institute, Wako, Japan

*** Correspondence:** Tetsuo Yamamori, RIKEN Brain Science Institute, Wako, Saitama 351-0198, Japan

E-mail: yamamori@nibb.ac.jp and tetsuo.yamamori@riken.jp


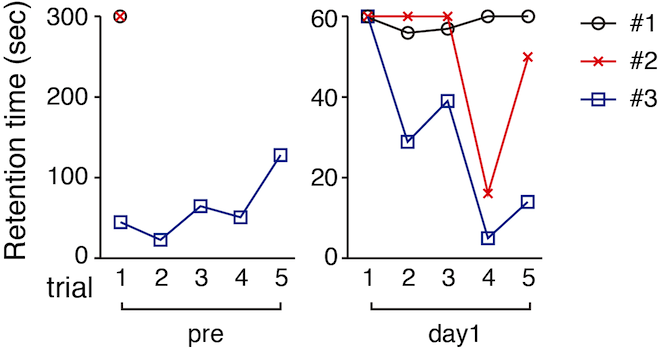


**Supplementary Figure 1: Performance of rota-rod task in Experiment 1.**

**(A)** In Experiment 1, mice were placed on the stationary (0 rpm) rota-rod on pre day. On the pre day, the mice were placed for 300 sec. If they fell, they were returned for a total 300 sec. **(B)** On the next day (day 1), they ran on the rotating rota-rod (10 rpm) for 60 sec up to 5 trials.


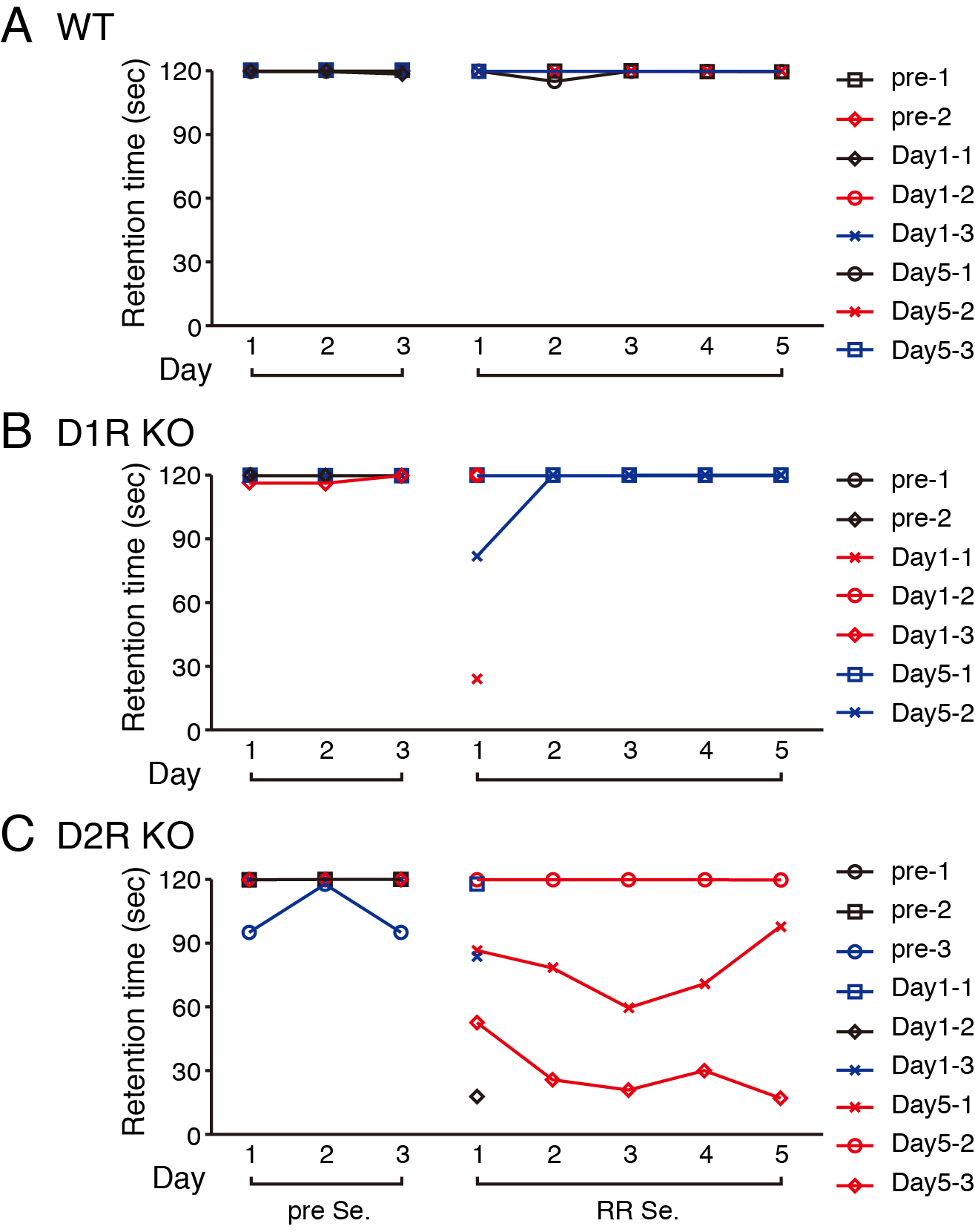


**Supplementary Figure 2: Performance of rota-rod task in Experiment 2.**

The performances of rota-rod task by **(A)** WT, **(B)** D1R KO, and **(C)** D2R KO mice in Experiment 2 are shown. Mice were placed on the stationary (0 rpm) rota-rod for the presession groups for 3 days and on the rotating (5 rpm) rota-rod for the rota-rod session groups. Mice were randomly divided into the pre, day 1 and day 5 groups.


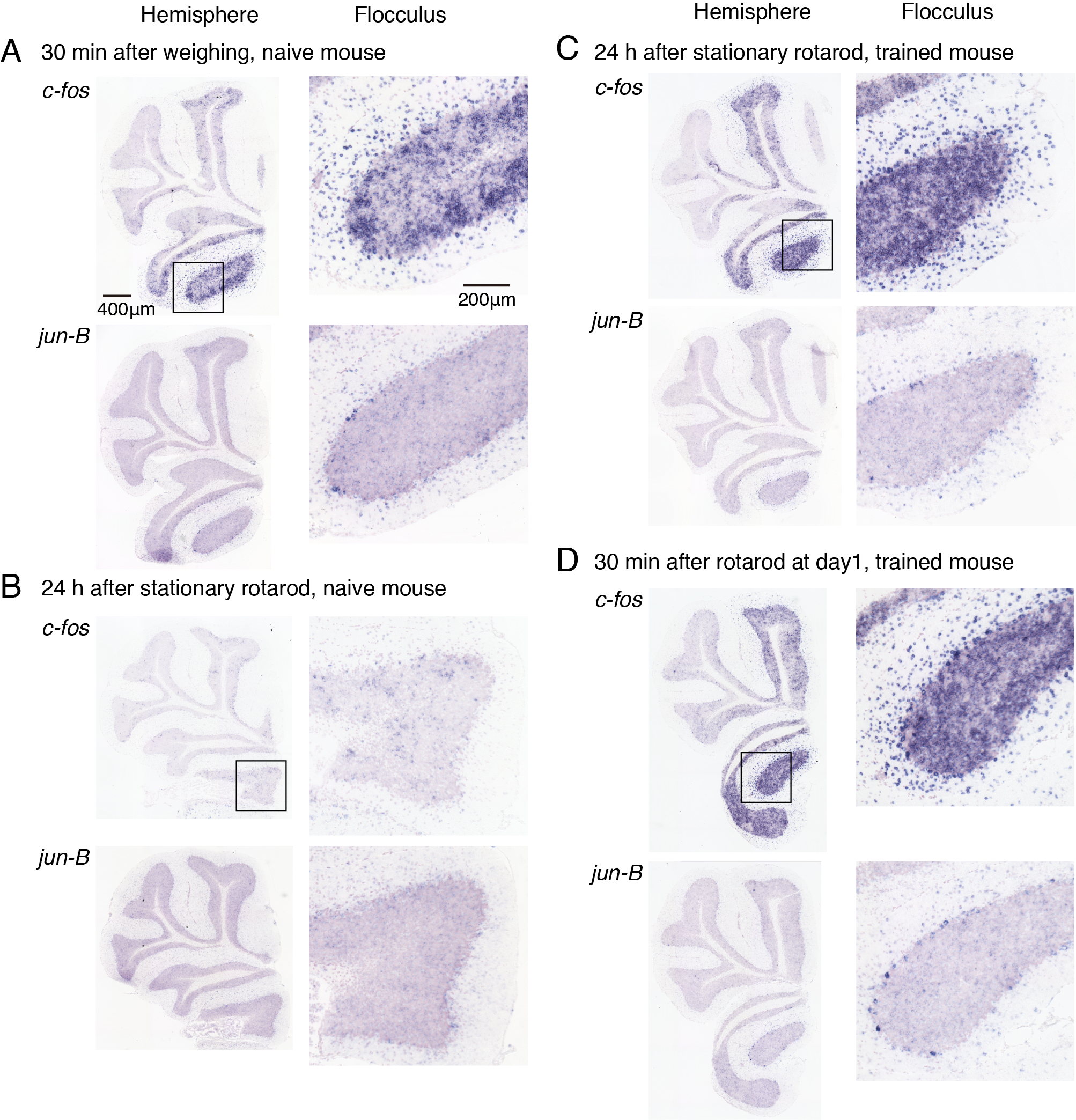


**Supplementary Figure 3: Expression levels of *c-fos* and *jun-B* mRNAs in the cerebellum of naive mice 30min after weighing or 24 h after stationary rota-rod.**

Mice that did not experience any motor tasks (untrained) were sacrificed at **(A)** 30 min after weighing (same section used in figure 7C) or **(B)** 24 h after the stationary rota-rod. This ISH staining was done at different place (at Osaka University). To compare these figures, we stained sections from the same mice used **(C)** in figure 8A (trained mouse, 24 h after stationary rotarod) and **(D)** in figure 9A (trained mouse, rota-rod task on day 1) at the same time. Right panels show magnified images of floccular signals.


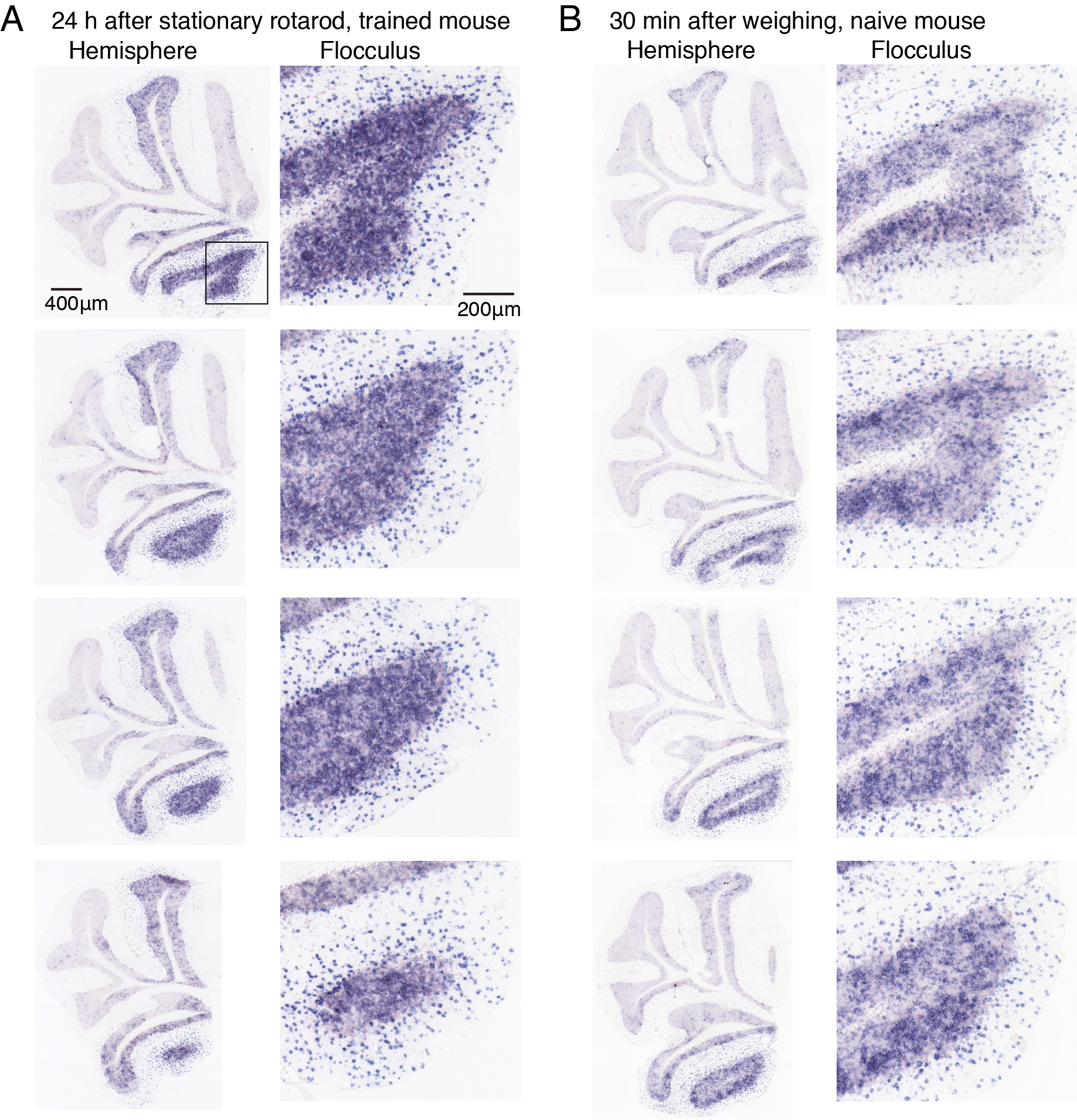


**Supplementary Figure 4:** **Expression pattern of *c-fos* in the cerebella of naive mice 30 min after single weighing and 24 h after stationary rota-rod**

*c-fos* expression was examined in a series of cerebellar sections. (A) 24 h after stationary rota-rod in the trained mouse shown in Figure 8A.  (B) naive mouse (the same mouse was used shown in figure 7C and supplementary Figure 3A). Both mice were sacrificed 30 min after weighing. The flocculus part as shown in a box of left panel was enlarged in each section of the right panel.


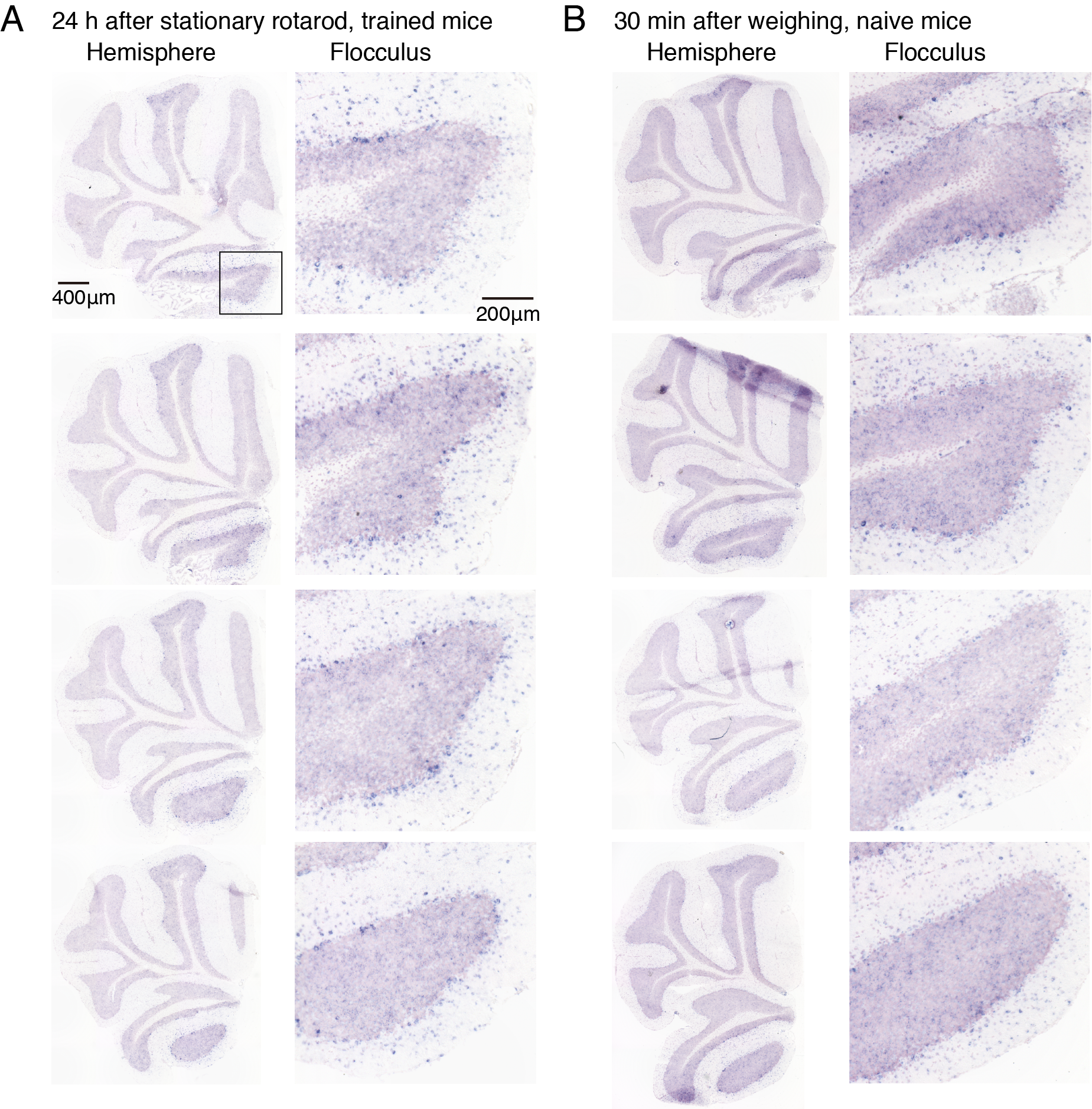


**Supplementary Figure 5:** **Expression pattern of *jun-B* in the cerebella of naive mice 30 min after single weighing and 24 h after stationary rota-rod**

*jun-B* expression was examined in a series of cerebellar sections. (A) 24 h after stationary rota-rod in the trained mouse shown in Figure 8A.  (B) naive mouse (the same mouse was used shown in figure 7C and supplementary Figure 3A). Both mice were sacrificed 30 min after weighing. The flocculus part as shown in a box of left panel was enlarged in each section of the right panel.

**Supplementary Table 1: Information on ISH probes used in this study.**

| Probe name | Position | Reference |
| --- | --- | --- |
| *Arc* | 1238 - 1928 | NM_001276684.1 |
| *c-fos* | 1-1291 | NM_010234.2 |
| *c-jun* | 899 - 1922 | NM_010591.1 |
| *fos-B* | 317 - 1149 | XM_006539544.1 |
| *jun-B* | 319 - 1359 | NM_008416.1 |
| *jun-D* | 1317 - 2291 | NM_001286944.1 |
| *krox 20 (Egr2)* | 287 - 1338 | XM_006513213.1 |
| *zif-268 (Egr1)* | 284 - 1866 | NM_007913.5 |

**Supplementary Chart 1: The list of motor tasks in experiment 2.**

The mice used in experiment 2 were experienced several sessions of motor tasks. D1R KO and D2R KO mice performed the same schedule (left). They were divided into two groups and performed different schedules during 6th to 12th week for Step-Wheel tasks. WT mice performed the other schedule (right).
